# A Grover-Like Skin Rash: A Potential Indicator of Underlying Severe Acute Respiratory Syndrome Coronavirus 2 (SARS-CoV-2) Infection

**DOI:** 10.7759/cureus.60173

**Published:** 2024-05-12

**Authors:** Gita Mihelcic, Tjaša Furlan, Hayder N Alhameedi, Samuel E Audet, Boštjan Leskovar

**Affiliations:** 1 Internal Medicine, District General Hospital Trbovlje, Trbovlje, SVN; 2 Internal Medicine, Mid and South Essex NHS Foundation Trust, Southend, GBR; 3 Dermatology, Mid and South Essex NHS Foundation Trust, Southend, GBR

**Keywords:** acantholysis, transient acantholytic dermatosis, covid-19, covid, sars-cov-2 infection, grover’s disease

## Abstract

Transient acantholytic dermatosis, also known as Grover’s disease, is an acquired dermatological condition characterised by the sudden emergence of pruritic, erythematous papules, or vesicles, primarily affecting the trunk. It is observed most commonly in men older than 50 years. Histology typically demonstrates a pattern of focal acantholysis within the epidermis, dyskeratotic cells including corps ronds and grains, and a variable perivascular lymphocytic infiltrate in the upper dermis. While its aetiology is not well understood, recognised triggers include excessive heat, sweating, sun exposure, and certain drugs, such as chemotherapy agents. More recently, isolated reports of Grover's disease and Grover-like skin eruptions have been described in patients with severe acute respiratory syndrome coronavirus 2 (SARS-CoV-2) infection and following COVID-19 vaccination. We report the case of a 65-year-old man who presented to secondary care with a nine-day history of an intensely pruritic rash over his chest and back. On internal medical workup, he was found to have SARS-CoV-2 infection and rapidly deteriorated due to coronavirus disease 2019 (COVID-19)-associated pneumonia, necessitating a 10-day hospital admission for supportive care. Diagnostic workup of his skin lesions confirmed transient acantholytic dermatosis (Grover's disease), which resolved following a course of oral corticosteroids. This case underscores the rare but significant association between Grover's disease and COVID-19, contributing valuable insights to the evolving body of literature on cutaneous lesions associated with SARS-CoV-2 infection, and highlighting the importance of considering SARS-CoV-2 screening as part of the diagnostic workup for patients presenting with Grover-like skin eruptions.

## Introduction

First described in 1970 by Ralph W. Grover, transient acantholytic dermatosis, commonly known as Grover’s disease, manifests as intensely pruritic oedematous papules, papulovesicles, or papulosquamous eruptions predominantly over the trunk and central chest and back [1]. Histologically characterised by a pattern of acantholysis and dyskeratosis within the epidermis, this condition predominantly affects Caucasian males over 50 years of age [2,3]. Environmental triggers such as heat and sweating are known exacerbating factors, and certain cases have been exacerbated by cold conditions. Additionally, systemic diseases or neurovegetative disruptions affecting sweat secretion may influence the disease's course [4]. The role of medications, especially chemotherapeutic agents such as docetaxel, etoposide, and daunorubicin, as well as aromatase inhibitors such as letrozole, and BRAF inhibitors have been implicated as potential triggers in drug-induced cases of Grover’s disease [5,6]. Recent evidence suggests a possible link between Grover’s disease and systemic infections such as severe acute respiratory syndrome coronavirus 2 (SARS-CoV-2), highlighting significant public health implications in the context of the coronavirus disease 2019 (COVID-19) pandemic [7]. Herein, we report a case of a patient presenting with a symptomatic Grover-like rash who was subsequently diagnosed with SARS-CoV-2 infection and experienced rapid health deterioration. We discuss the broader implications of these findings within the context of cutaneous lesions associated with SARS-CoV-2 and their impact on global healthcare practices.

## Case presentation

**Figure 1:**
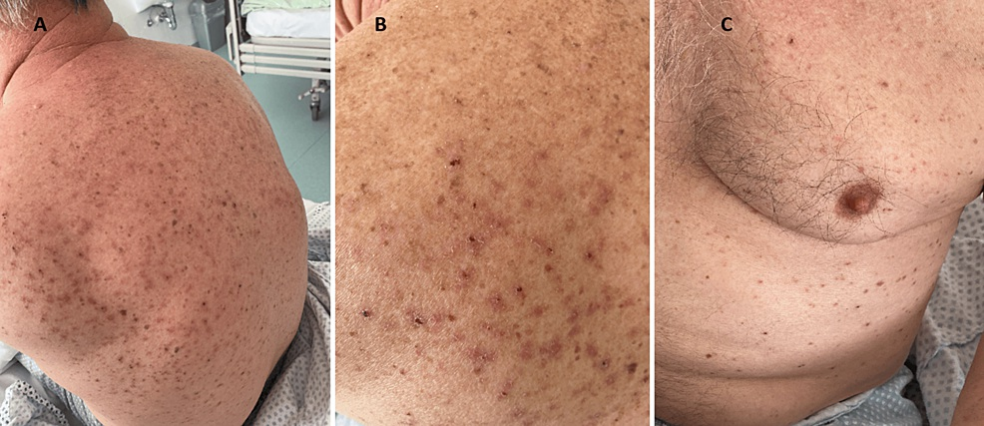
A and B: Eroded erythematous to violaceous papules distributed across the back. C: Continuation of lesions over the chest and abdomen. The surrounding skin is clear.

A 65-year-old Caucasian man presented to the Acute Medical Unit with a nine-day history of an intensely pruritic rash on his back and chest, and no other associated symptoms. His past medical history was significant only for hypertension, for which he took perindopril, and he had no known drug allergies and took no over-the-counter medications or supplements. On examination, the patient was afebrile and normotensive but notably hypoxic on room air with an oxygen saturation of 90% and tachycardic with a heart rate of 120 beats per minute. Dermatological examination revealed widespread eroded erythematous papules visible over the patient’s back and chest (Figure 1).

There was no history of dyspnoea, chest pain, or abdominal, joint, or muscle pain. It was noted, however, that he had declined vaccination against SARS-CoV-2 due to personal beliefs. Initial work-up revealed raised inflammatory markers but otherwise unremarkable blood tests and type 1 respiratory failure on an arterial blood gas (Table 1). An erect posteroanterior chest X-ray showed bilateral peripheral ground-glass opacities. A respiratory viral screen was performed, which returned positive for SARS-Cov-2 infection. As treatment for COVID-19, the patient was commenced on intravenous methylprednisolone and prophylactic trimethoprim and sulphamethoxazole.

**Table 1:** Initial blood test results on admission

Description	Result	Normal Values
Haemoglobin (Men)	171 g/L	130-170 g/L
White Blood Cells	6.96 x 10^9/L	4.0-10.0 x 10^9/L
Platelets	182 x 10^9/L	150-410 x 10^9/L
Neutrophils	5.43 x 10^9/L	1.5-7.4 x 10^9/L
Lymphocytes	0.78 x 10^9/L	1.1-3.5 x 10^9/L
Eosinophils	0.0 x 10^9/L	0.02-0.67 x 10^9/L
Basophils	0.01 x 10^9/L	0.00-0.13 x 10^9/L
Erythrocyte Sedimentation Rate (ESR)	28 mm/h	0-15 mm/hr
Aspartate Aminotransferase (AST)	53 U/L	3-38 U/L
Alanine Transaminase (ALT)	33 U/L	3-43 U/L
Alkaline Phosphatase (ALP)	42 U/L	30-120 U/L
Total Bilirubin	20 µmol/L	0-17 µmol/L
Urea	9.4 mmol/L	2.8-7.5 mmol/L
Creatinine	113 µmol/L	44-97 µmol/L
Sodium	136 mmol/L	135-145 mmol/L
Potassium	4.5 mmol/L	3.8-5.5 mmol/L
C-Reactive Protein (CRP)	135 mg/L	< 5 mg/L
Arterial pH	7.44	7.36-7.42
Arterial pCO2	4.5 kPa	4.9-5.9 kPa
Arterial pO2	7.0 kPa	10.6-13.3 kPa
Arterial HCO3	22.1 mmol/L	22-30 mmol/L
Arterial Base Excess	-1.1 mmol/L	-2.3 to +2.3 mmol/L

**Figure 2:**
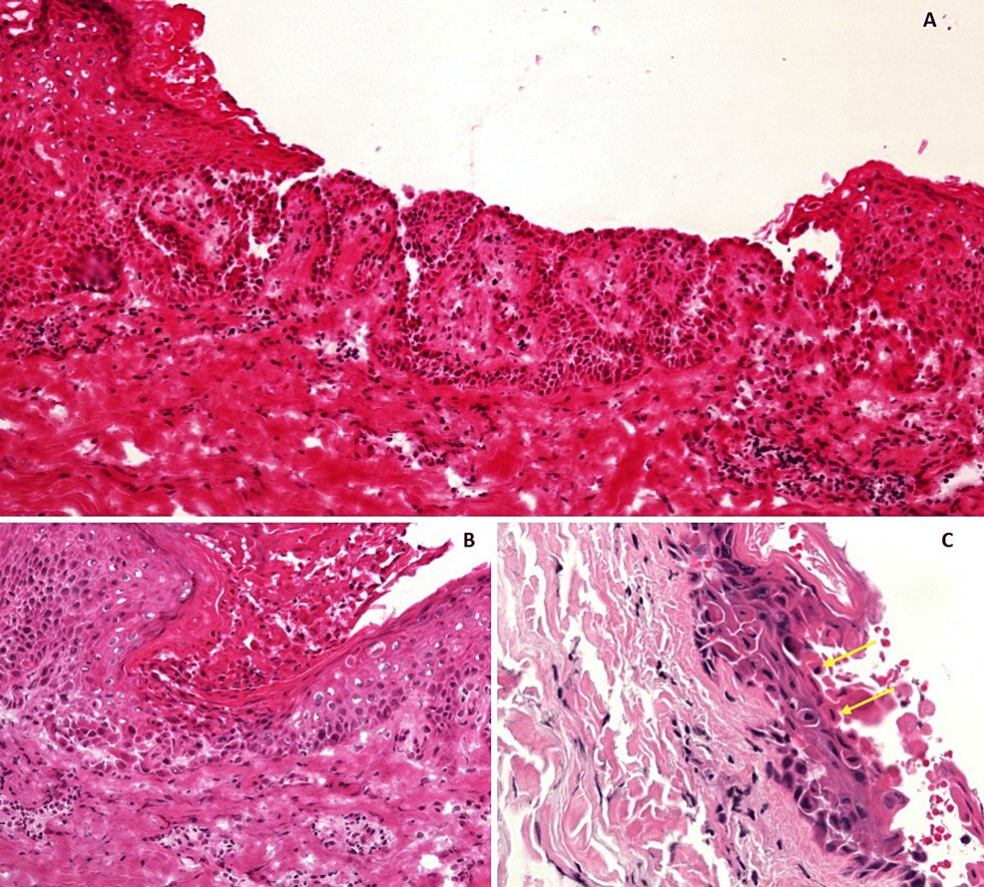
Haematoxylin and eosin (H&E) staining at three different magnifications, 100x (A), 200x (B), and 400x (C). All three images depict acantholysis. Image B additionally demonstrates dermal lymphocytic infiltration. Image C demonstrates dyskeratosis with the presence of corps ronds (yellow arrows) with eosinophilic cytoplasm and central pyknotic nuclei, amidst separated keratinocytes.

Bacterial and viral swabs were taken from his skin lesions were unremarkable. There was no evidence of Herpes Simplex Virus Type 1 (HSV-1), Herpes Simplex Virus Type 2 (HSV-2), and varicella-zoster virus (VZV). Skin biopsies were taken from lesions on the back, with histopathological analysis revealing suprabasal acantholysis, dyskeratosis, corps ronds, and lymphocytic infiltration in the upper layers of the dermis, with no evidence of vasculitis (Figure 2). Direct immunofluorescence was unremarkable. This was consistent with a diagnosis of Grover’s disease.

As the patient had already commenced corticosteroids for his SARS-CoV-2 infection, it was decided not to introduce additional treatment for his skin lesions but to monitor their progression. Over the next 10 days of his hospital stay, there was a significant improvement in the severity of the skin lesions, and the patient reported a notable reduction in pruritus. Following this improvement, he was discharged on a tapering course of oral methylprednisolone.

One month following discharge, the patient presented to the outpatient medical day clinic for review, having completed the weaning course of oral methylprednisolone. The skin lesions had fully resolved, and he reported no further pruritus or any COVID-19-related symptoms. Consequently, it was determined that no additional follow-up would be necessary from either a general medical or dermatological perspective. The patient was subsequently discharged back to primary care.

## Discussion

The underlying aetiopathology of Grover’s disease is not well understood. However, several potential exacerbating factors have been described, including febrile illness, prolonged bed rest, exposure to extremes of temperature, dry air, and radiation. Grover’s disease has also been reported in association with several medical conditions and treatments, including malignancy, chemotherapy induction, chronic renal failure, and the commencement of hemodialysis [2,3,8]. Differential diagnosis is crucial, as Grover’s disease can mimic other acantholytic disorders, including pemphigus vulgaris, Darier's disease (keratosis follicularis), Hailey-Hailey disease (benign familial pemphigus), and pemphigus foliaceus, as well as other dermatological entities such as miliaria and dermatitis herpetiformis [4,9,10]. Definitive diagnosis relies on histopathological examination of a skin biopsy, where up to four histological patterns can be identified: Hailey-Hailey-like, Darier-like, spongiotic, and pemphigus-like [2]. Direct immunofluorescence may also be performed to rule out autoimmune blistering diseases. In cases where Grover-like skin lesions appear extensively or in atypical distributions beyond the trunk and extremities, there is some evidence to suggest an increased association with underlying malignancy [11]. Clinicians should consider a comprehensive internal investigation to exclude the potential underlying malignancies when encountering such extensive or atypically distributed Grover-like skin lesions.

In 2020, a Grover-like skin eruption was reported in a patient with SARS-Cov-2 infection, with antibodies to the SARS‐Cov‐2 spike protein detected in the patient’s endothelium and eccrine sweat gland epithelium [7]. Since then, there have been further isolated reports of transient acantholytic dermatosis and Grover-like skin eruptions occurring in patients with SARS-CoV-2 infection.

Cutaneous lesions associated with SARS-CoV-2 infection are commonly observed in clinical practice, with their prevalence estimated to be around 6% [12]. Although no formal classification has yet been established, the cutaneous manifestations associated with SARS-CoV-2 infection can generally be categorised into six groups (Table 2) [13].

**Table 2:** Cutaneous manifestations associated with SARS-CoV-2 infection

Cutaneous manifestations	Description
Maculopapular eruptions	are the most common dermatological manifestation of COVID-19, predominantly observed in middle-aged individuals, with lesions typically located on the trunk
Urticarial eruptions	manifest as hives or angioedema, characterised by erythematous, intensely pruritic rashes, usually appearing on the trunk and/or limbs
Vesicular eruptions	are less frequent, affecting middle-aged individuals, often presenting on the trunk and potentially spreading with polymorphic lesions to the extremities
Petechiae/purpura lesions	present usually in middle-aged individuals after COVID-19 symptoms, often presenting on the trunk and extremities
Chilblain-like lesions (CBLLs)	also known as 'COVID toes,' emerge as late manifestations, often bullous, predominantly affecting children and young adults. Unlike typical chilblain responses to cold exposure; these localised inflammatory disorders result in swelling, erythema, and violaceous colouration on extremities
Livedo or necrosis	is characterised by a livedo reticularis-like pattern; these eruptions are among the rarer dermatological associations of COVID-19, primarily observed in severe cases among the elderly. These lesions appear on the trunk and extremities and are linked to the highest mortality rate among COVID-19-related skin manifestations

Other cutaneous lesions associated with SARS‐CoV‐2 infection include pityriasis rosea, sebopsoriasis, lichen planus, onychopathy, pruriginous nodules, eruptive angiomas, and notably, Grover-like skin eruptions [7,13].

Both Grover-like skin eruptions and histopathological confirmed Grover’s disease have been reported in patients with SARS-CoV-2 infection since 2020, supporting the potential link between SARS‐Cov‐2 infection and transient acantholytic dermatosis [7,14]. Interestingly, Grover-like skin eruptions have also been observed following the administration of COVID-19 vaccines, with reports emerging of such reactions in three individuals - globally two after receiving the Moderna (mRNA-1273) vaccine and one post-administration of the Pfizer COVID-19 vaccine [14-16]. The exact mechanisms underlying these dermatological manifestations are not fully understood and could be multifaceted, including direct viral effects on the skin cells, immune-mediated reactions, or other indirect systemic responses such as stress or fever secondary to infection or vaccination. Further research is needed to clarify these pathways and to establish a definitive causal relationship between Grover's disease and COVID-19 infections, which will help in developing targeted treatments and management strategies.

The management of Grover's disease primarily focuses on symptom control. While the condition can be self-limiting, it can also persist for months to years, which can be challenging to control. First-line treatments include the application of topical corticosteroids to reduce inflammation and itch, alongside moisturisers to alleviate skin dryness. For patients experiencing intense pruritus, antihistamines may provide additional relief. In more severe or refractory cases, systemic therapies such as oral retinoids (e.g. acitretin) or corticosteroids may be considered [2]. Phototherapy, specifically narrow-band ultraviolet B (NB-UVB) light, has been effective in some patients. Blue light phototherapy has also shown to be a safe and effective treatment modality for patients with Grover's disease [17]. Environmental modifications such as reducing heat exposure, minimising sweating, and avoiding known triggers are also recommended to help manage symptoms and prevent exacerbations of Grover's disease. Due to the potential for relapse, a long-term management plan may be necessary, and collaboration with a dermatologist is crucial for complex cases or when first-line therapies fail.

## Conclusions

Grover’s disease, or transient acantholytic dermatosis, is an acquired papulovesicular skin disorder with distinct clinical and histopathological features, yet a poorly understood aetiology. While the condition responds in most cases to standard dermatological treatments, its presence in the context of COVID-19 necessitates a careful evaluation. Our findings underscore the potential utility of including SARS-CoV-2 screening in the diagnostic work-up for patients presenting with dermatological symptoms typical of Grover’s disease, particularly when no other obvious triggers are present. This approach could lead to more timely and precise management of COVID-19, particularly in patients at higher risk of severe outcomes. Our report contributes to the wider and evolving body of literature on cutaneous lesions associated with SARS-CoV-2 infection, although further investigation into the relationship between Grover's disease and SARS-CoV-2 infection is needed to establish the underlying pathophysiological mechanisms and inform more targeted management strategies.
